# 

**Published:** 1891-06

**Authors:** 


### Society Meetings.

The Medical Association of Western New York.—The twenty-fourth annual meeting of the Medical Association of Central New York will be held in the Lecture Hall of the Y. M. C. A. Building, Mohawk and Pearl streets, Buffalo, N. Y., on Tuesday, June 2d, at 10 o'clock, A. M. The provisional programme comprises the following papers:

PRESIDENT'S ADDRESS.

The Converging Lines of Medical and Surgical Treatment, by Nathan Jacobson, M. D., Syracuse, N. Y.

GYNECOLOGICAL.

Operative Treatment of Retroflexion of the Uterus, by M. D. Mann, M. D., Buffalo, N. Y.; Cases Illustrative of the Difficulty of Diagnosticating Abdominal and Pelvic Tumors, by John Van Duyn, M. D., Syracuse, N. Y.; Ill Effects of Electricity upon large Uterine Fibroids, by C. O. Baker, M. D., Auburn, N. Y.; Ectopic Gestation, by Henry D. Ingraham, Buffalo, N. Y.

SURGICAL.

Discussion on Epilepsy: Pathology, by Jas. W. Putnam, M. D., Buffalo, N. Y.; Effects on Peripheral Irritation in Inducing It, by Dr. Wm. C. Krauss, Buffalo, N. Y.; Medical Treatment, by Floyd S. Crego, M. D., Buffalo, N. Y.; Surgical Aspects, by Roswell Park, M. D., Buffalo, N. Y.; Osteo-Sarcoma, by H. W. Carpenter, M. D., Oneida, N. Y.; Arthrectomy of the Knee Joint, by Herman Mynter, M. D., Buffalo, N. Y.; Operative Treatment of Spastic Paralysis, by L. A. Weigel, M. D., Rochester, N. Y.; Report of a Case of Unusual Fracture of the Inferior Maxilla, by Wm. C. Barrett, M. D., Buffalo, N. Y.

MEDICAL.

Treatment of Pulmonary Hemorrhage, by N. W. Soble, M. D., Rochester, N. Y.; Pneumonia as I See and Treat It, by A. A. Young, M. D., Newark, N. Y.; Traumatic Hysteria from Railroad Injury, by J. B. Andrews, M. D., Buffalo, N. Y.; Traumatic Electric Neurosis, by Jas. W. Putnam, M. D., Buffalo, N. Y.; Acute Chorea in a Man Sixty-eight Years old, by W. R. Howard, M. D., Rochester, N. Y.; Rhus Aromatica in Incontinentia Urinæ, by W. C. Krauss, M. D., Buffalo, N. Y. The Clinical and Pathological Significance of Vertigo, by Henry L. Elsner, M. D., Syracuse, N. Y.; Vertigo and the Eyes, by Lucien Howe, M. D., Buffalo, N. Y.; the Therapeutic Indications offered by the Clinical and Pathological Study of Vertigo, by Floyd S. Crego, M. D., Buffalo, N. Y.; Virchow's Post-Mortems and the Tissues Especially Influenced by Tuberculin, by Wm. C. Bailey, M. D., Albion, N. Y.; Motor Disturbances of the Stomach, by Chas. G. Stockton, M. D., Buffalo, N. Y.; the Rational Treatment of Digestive Disorders, by Albert E. Persons, M. D., Buffalo, N. Y.; Hydrochloric Acid in Gastric Disease, by A. L. Benedict, M. D., Buffalo, N. Y.; Deficient Oxygenation as an Etiological Factor in Disease, by DeLancey Rochester, M. D., Buffalo, N. Y.; The Use of Oxygen for the Correction of Morbific Material in the Blood, by Samuel G. Dorr, M. D., Buffalo, N. Y.; Report of Rare Cases of Skin Disease, by Geo. J. Lund, M. D., Medina, N. Y.; Infantile Eczema, by Ernest Wende, M. D., Buffalo, N. Y.

THROAT AND NOSE.

Anenoid Growths of the Naso-Pharynx and their Relation to Aural Disease—Remarks on Treatment, by Jas. E. Nichols, M. D., New York City; Intubation in Syphilitic Stenosis of the Larynx, by John O. Roe, M. D., Rochester, N. Y.; Diagnosis of Throat and Nose Diseases, with Suggestions Regarding Some of the Minor Operations, by F. A. Mandeville, M. D., Rochester, N. Y.; Some After Effects of the Recent Influenza, by Frank H. Potter, M. D., Buffalo, N. Y.

EYE AND EAR.

*a* Ocular Reflexes; *b* Presentation of a Case of Hematoma Auris in a Sane Patient, by Alvin A. Hubbell, M. D., Buffalo, N. Y.; Influenza and Ear Disease, by J. Falk, M. D., Buffalo, N. Y.; *a* Formation of Bone in the Eye with Specimen; *b* Demonstration of an Instrument for Testing the Vision, by Lucien Howe, M. D., Buffalo, N. Y.

GENERAL.

Cremation, by J. C. Thompson, M. D., Buffalo, N. Y.; Electricity in General Practice, by Ira C. Brown, M. D., Buffalo, N. Y.; Overcrowding of Tenement Houses, by W. C. Callahan, M. D., Buffalo, N. Y.

At the Rochester meeting last year, by unanimous vote, the Erie County Society was elected to membership in the Association, and Buffalo appointed as the place of meeting for this year. It was also decided to discontinue the Autumn meeting, and hold the annual meeting in June, alternately at Buffalo, Syracuse and Rochester.

Luncheon will be served at "Oaks'," 64 W. Genesee street, at 12.30 P. M.

NATHAN JACOBSON, *President.*

EDWARD B. ANGELL, *Secretary.*

---

THE NATIONAL ASSOCIATION OF RAILWAY SURGEONS held a two days' meeting in Buffalo, commencing April 30th. About 150 members were present from various parts of the Union, chiefly from the south and west. A number of interesting papers were read and discussed. Dr. and Mrs. B. H. Daggett, of this city, gave a reception in honor of the delegates on Thursday evening, April 30th, which was largely attended and was a most enjoyable occasion. An excursion was given to the members to Niagara Falls, on Saturday, May 2d, after the final adjournment. Dr. Murphy, of St. Paul, was chosen President, and the next place of meeting is White Sulphur Springs, W. Va.

### BOOKS AND PAMPHLETS RECEIVED.

A Treatise on the Diseases of the Nervous System. By William A. Hammond, M. D., Surgeon-General U. S. Army (Retired List); late Professor of Diseases of the Mind and Nervous System in the college of Physicians and Surgeons of New York, the Bellevue Hospital Medical College, the University of the City of New York, and the New York Post-Graduate Medical School and Hospital, etc. With the collaboration of Græme M. Hammond, M. D., Professor of Diseases of the Mind and Nervous System in the New York Post-Graduate Medical School and Hospital, etc. With 118 illustrations. Ninth edition, with corrections and additions. 8vo, pp. 932. New York: D. Appleton & Company. 1891. Buffalo: Otto Ulbrich. Price, cloth, $5.00.

Materia Medica and Therapeutics, with especial reference to the Clinical Application of Drugs. By John V. Shoemaker, A. M., M. D., Professor of Materia Medica, Pharmacology, Therapeutics, and Clinical Medicine, and Clinical Professor of Diseases of the Skin in the Medico-Chirurgical College of Philadelphia; Physician to the Medico-Chirurgical Hospital, etc. Volume II. of a Treatise on Materia Medica, Pharmacology, and Therapeutics, being an independent volume upon drugs. 8vo, pp. xx.—652. Philadelphia and London: F. A. Davis. 1891. Price, cloth, $3.50; sheep, $4.50.

Medical Symbolism, in Connection with Historical Studies in the Arts of Healing and Hygiene. Illustrated. By Thomas S. Sozinskey, M. D., Ph. D., author of The Culture of Beauty, The Care and Culture of Children, etc. No. 9 in the Physicians' and Students' Ready Reference Series. Philadelphia and London: F. A. Davis. 1891.

Fever: Its Pathology and Treatment by Antipyretics. Boylston Prize Essay of Harvard University, July, 1890. By Robert Amory Hare, M. D., B. Sc., Clinical Professor of Diseases of Children and Demonstrator of Therapeutics in the University of Pennsylvania; Physician to St. Agnes Hospital and to the Children's Dispensary of the Children's Hospital, etc. No. 10 in the Physicians' and Students' Ready Reference Series. Philadelphia and London: F. A. Davis. 1891. Price, cloth, $1.25 net.

A Practical Treatise on Diseases of the Skin. By Henry G. Piffard, M. D., Clinical Professor of Dermatology, University of the City of New York; Surgeon in charge of the New York Dispensary for Diseases of the Skin; Consulting Surgeon to Charity Hospital, etc., etc. Assisted by Robert M. Fuller, M. D. With fifty full-page original plates, and thirty-three illustrations in the text. Large quarto, pp. vi.—159. New York: D. Appleton & Company. London: Caxton House, Paternoster Square. 1891. Price, $15.00.

The Pocket Materia Medica and Therapeutics; a Résumé of the Action and Doses of all Officinal and Non-officinal Drugs now in Common Use. By C. Henri Leonard, A. M., M. D., Professor of Medical and Surgical Diseases of Women and Clinical Gynecology in the Detroit College of Medicine. Cloth; 12mo, pp. 300. Price, postpaid, $1.00. The Illustrated Medical Journal Company, publishers, Detroit.

Surgical Bacteriology. By N. Senn, M. D., Ph. D., Professor of Surgery in Rush Medical College, Chicago, and in the Chicago Polyclinic; Attending Surgeon to the Milwaukee Hospital; Consulting Surgeon to the Milwaukee County Hospital and to the Milwaukee County Insane Asylum; Honorary Fellow College of Physicians of Philadelphia; Permanent Member of the German Congress of Surgeons; Corresponding Member Harveian Society, London; Honorary Member of La Academia de la Medicina de Mexico, etc. Second edition, thoroughly revised. Philadelphia: Lea Brothers & Co. 1891.

International Clinics: A Quarterly of Clinical Lectures on Medicine, Surgery, Gynecology, Pediatrics, Neurology, Dermatology, Laryngology, Ophthalmology, and Otology. By professors and lecturers in the leading medical colleges of the United States, Great Britain, and Canada. Edited by John M. Keating, M. D., Philadelphia; J. P. Crozer Griffith, M. D., Philadelphia; J. Mitchell Bruce, M. D., F. R. C. P., London, Eng.; and David W. Finlay, M. D., F. R. C. P., London, Eng. April, 1891. Large octavo; pp. viii.—350. Illustrated. Philadelphia: J. B. Lippincott Company. Price, cloth, $2.75; half leather, $3.00.

Origin, Purpose, and Destiny of Man; or, The Philosophy of the Three Ethers. By William Thornton. 12mo, pp. 100. Boston: Published by the author. 1891.

Seventeenth Annual Report of the Secretary of the State Board of Health of the State of Michigan for the fiscal year ending June 30, 1889. Lansing, Mich.: Darius D. Thorp, Printer and Binder. 1890.

Vesico-Vaginal Fistula. With some Aberrant Cases illustrating the Cause of Leakage after Successful Closure of the Fistula—Vesical Calculi in connection with Fistula—Utero-vaginal Fistula. By Henry Fraser Campbell, M. D., Augusta, Ga. Reprint: Transactions Southern Surgical and Gynecological Association. 1890.

Practical Notes on Urinary Analysis. By William B. Canfield, A. M., M. D., Chief of Chest Clinic and Lecturer on Clinical Medicine, University of Maryland; Visiting Physician to the Union Protestant Infirmary, Bay View Hospital, Baltimore, etc. Physicians' Leisure Library, Detroit, Mich.: George S. Davis. 1891. Price, cloth, 50 cents; paper, 25 cents.

Cosmetics. A Treatise for Physicians and Pharmacists. By Dr. Heinrich Paschkis, Docent at the University of Vienna. 8vo, pp. 210; paper. New York: William Wood & Company. 1891. Price, $1.50.

Eleventh Inaugural Address of Clark Bell, Esq., President of the Medico-Legal Society, New York. Reprint: *The Medico-Legal Journal*, December, 1890.

Cornell University Agricultural Experiment Station. Bulletin 26. Horticultural Division, March, 1891. Experiences with Egg Plants. Ithaca: Published by the University. 1891.

